# Pharmacogenetic Aspects of the Interaction of AT1 Receptor Antagonists With ATP-Binding Cassette Transporter *ABCG2*

**DOI:** 10.3389/fphar.2018.00463

**Published:** 2018-05-14

**Authors:** Anne Ripperger, Anna Krischer, Dina Robaa, Wolfgang Sippl, Ralf A. Benndorf

**Affiliations:** ^1^Department of Clinical Pharmacy and Pharmacotherapy, Institute of Pharmacy, Martin-Luther-University Halle-Wittenberg, Halle, Germany; ^2^Department of Medicinal Chemistry, Institute of Pharmacy, Martin-Luther-University Halle-Wittenberg, Halle, Germany

**Keywords:** ATP-binding cassette (ABC) transporter, ABCG2, AT1 receptor antagonists, F489L polymorphism, pharmacogenetics

## Abstract

The ATP-binding cassette transporter ABCG2 (BCRP and MXR) is involved in the absorption, distribution, and elimination of numerous drugs. Thus, drugs that are able to reduce the activity of ABCG2, e.g., antihypertensive AT1 receptor antagonists (ARBs), may cause drug-drug interactions and compromise drug safety and efficacy. In addition, genetic variability within the ABCG2 gene may influence the ability of the transporter to interact with ARBs. Thus, the aim of this study was to characterize the ARB-ABCG2 interaction in the light of naturally occurring variations (F489L, R482G) or amino acid substitutions with *in silico*-predicted relevance for the ARB-ABCG2 interaction (Y469A; M483F; Y570A). For this purpose, ABCG2 variants were expressed in HEK293 cells and the impact of ARBs on ABCG2 activity was studied *in vitro* using the pheophorbide A (PhA) efflux assay. First, we demonstrated that both the F489L and the Y469A substitution, respectively, reduced ABCG2 protein levels in these cells. Moreover, both substitutions enhanced the inhibitory effect of candesartan cilexetil, irbesartan, losartan, and telmisartan on ABCG2-mediated PhA efflux, whereas the R482G substitution blunted the inhibitory effect of candesartan cilexetil and telmisartan in this regard. In contrast, the ARB-ABCG2 interaction was not altered in cells expressing either the M483F or the Y570A variant, respectively. In conclusion, our data indicate that the third transmembrane helix and adjacent regions of ABCG2 may be of major importance for the interaction of ARBs with the ABC transporter. Moreover, we conclude from our data that individuals carrying the F489L polymorphism may be at increased risk of developing ABCG2-related drug-drug interactions in multi-drug regimens involving ARBs.

## Introduction

In humans, at least 48 distinct ATP-binding cassette (ABC) transporters have been described that are divided into seven subfamilies (A–G) based on structural similarities and sequence homology (Moitra and Dean, 2011). The ABC transporter ABCG2, also known as breast cancer resistance protein (BCRP) or mitoxantrone resistance-associated protein (MXR), was cloned independently in 1998 from both a drug-selected human breast cancer cell line and a human cDNA library (Allikmets et al., 1998; Doyle et al., 1998). It is a ‘half transporter’ consisting of one hydrophilic nucleotide binding domain (NBD) located in the cytoplasm and one hydrophobic membrane-spanning domain (MSD), whereas the functional form of ABCG2 is a homodimer with a molecular mass of approximately 144 kDa (Taylor et al., 2017). Besides playing an important role in multidrug resistance of cancer cells, ABCG2 is expressed, i.e., in the apical membrane of intestinal epithelial cells, the canalicular membrane of hepatocytes, and at the placental-fetal interface, where it reduces uptake and tissue-specific disposition of drugs that serve as substrates of the ABC transporter (Allikmets et al., 1998; Ni and Mao, 2011; Zolk and Fromm, 2011; Stacy et al., 2013; Schumacher and Benndorf, 2017). On the other hand, ABCG2-mediated transport (and elimination) of substrate drugs may be reduced by concomitant application of drugs that inhibit ABCG2 activity. Thus, drug-induced inhibition of ABCG2 activity may cause drug-drug-interactions, i.e., increased drug exposure and altered tissue disposition, by reducing ABCG2-mediated elimination of substrate drugs at above-mentioned sites. In this context, single nucleotide polymorphisms (SNPs) or acquired somatic mutations of ABCG2, e.g., the gain of function variant R482G that has been observed in multidrug-resistant cancer cells, may affect the interaction of drugs with the transporter and thus influence drug distribution as well as drug response and the incidence of drug-drug interactions. Indeed, several SNPs and somatic mutations in the *ABCG2* gene have been described which may contribute to the considerable pharmacokinetic and pharmacodynamic variability of drugs that are transported by ABCG2 (Erdem et al., 2012; Ieiri, 2012).

AT1 receptor antagonists (ARBs) represent a heavily prescribed group of first-line antihypertensive drugs that are also of major importance in the treatment of patients suffering from heart failure or diabetic nephropathy (Deppe et al., 2010). We have previously demonstrated that the ARBs telmisartan and candesartan cilexetil can act as relevant inhibitors of ABCG2 function, at least *in vitro* (Weiss et al., 2010; Deppe et al., 2014). However, candesartan cilexetil represents a prodrug that is rapidly converted to its active metabolite, candesartan, after intestinal absorption *in vivo* and, thus, most likely only has the potential to affect the function of ABCG2 expressed at the luminal side of intestinal epithelial cells. As ARBs are frequently used in multi-drug regimens involving ABCG2 substrates, ARB-induced inhibition of ABCG2 function may contribute to clinically relevant drug-drug interactions and altered drug disposition, thereby potentially compromising drug safety and efficacy. Moreover, in previous investigations we were able to demonstrate that genetic variability in the *ABCG2* gene influences the 3′UTR-mediated regulation of ABCG2 expression and was also able to affect the interaction of the ARB telmisartan with the ABC transporter (Deppe et al., 2014; Ripperger and Benndorf, 2016). In this regard, we could show that the F489L polymorphism largely increased telmisartan-induced inhibition of ABCG2-mediated pheophorbide A (PhA) transport apparently both in terms of inhibitory potency and efficacy *in vitro*, while the R482G variant reduced the inhibitory effect of telmisartan in this experimental system (Deppe et al., 2014). Thus, the aim of this study was to elucidate the impact of the F489L polymorphism and the R482G substitution on the interaction of ABCG2 with further ARBs known to affect the efflux function of ABC transporters (Weiss et al., 2010), i.e., candesartan cilexetil, irbesartan, and losartan. Moreover, we performed *in silico* ABCG2 homology modeling and molecular interaction analyses of selected ARBs to predict amino acid residues with relevance for the ARB-ABCG2 interaction and subsequently validated the impact of several amino acid substitutions (Y469A; M483F; Y570A) identified by the *in silico*-predicted relevance model using expression of ABCG2 variants in HEK293 cells and the PhA efflux assay *in vitro*. We used homology modeling on the basis of the mouse P-glycoprotein (ABCB1) (Aller et al., 2009), since the crystal structure of ABCG2 was not available when we started our *in silico* studies. Only recently, the complete structure of human ABCG2 in complex with two antigen-binding fragments of 5D3 (5D3-Fab) was successfully determined (Taylor et al., 2017), which provided a far more accurate insight into the structure of this transporter. This structure was, hence, used to rationalize the herein obtained *in vitro* results and to further study the molecular interactions of ARBs by docking studies.

## Materials and Methods

### Materials

Unless stated otherwise, chemicals were purchased from Sigma-Aldrich (Taufkirchen, Germany). Cell culture media and transfection reagents were bought from Thermo Fisher Scientific (Waltham, MA, United States). The pTRE-Tight-BI-AcGFP1 vector was from Clontech (Mountain View, CA, United States) and doxycycline was obtained from AppliChem (Darmstadt, Germany). Oligonucleotides were from Eurofins MWG Operon (Ebersberg, Germany). Pheophorbide A (PhA) was from Frontier Scientific Europe (Carnforth, Lancashire, United Kingdom). The AT1 receptor antagonists (ARBs) candesartan cilexetil, irbesartan, losartan, and telmisartan as well as the dihydropyridine calcium channel blocker nisoldipine were obtained from Sequoia Research Products (Pangbourne, United Kingdom).

### Site-Directed Mutagenesis

For expression of human ABCG2 (NM_004827) in HEK293 Tet-On cells, the ABCG2 coding sequence was transferred to the pTRE-Tight-BI-AcGFP1 vector as previously described and validated (Deppe et al., 2014). The pTRE-Tight-BI-AcGFP1-ABCG2 vector structure is depicted in **Supplementary Figure S1**. Naturally occurring ABCG2 variations R482G and F489L as well as amino acid substitutions with *in silico* predicted relevance for the ARB-ABCG2 interaction (Y469A, M483F, Y570A) were inserted into the ABCG2 cDNA sequence in the pTRE-Tight-BI-AcGFP1-ABCG2 plasmid using the QuickChange^®^ Lightning Site-Directed Mutagenesis Kit (Agilent Technologies, Waldbronn, Germany) with specific primers according to the manufacturer’s instructions.

The sense primers were synthesized as follows:

Y469A: 5′-GGATACTACAGAGTGTCATCT**GC**TTTCCT TGGAAAACTGTTATC-3′,
R482G: 5′-GATTTATTACCCATG**G**GGATGTTACCAAG T-3′,
M483F: 5′-CTGATTTATTACCCATGAGG**T**T**C**TTACCAA GTATTATATTTAC-3′,
F489L: 5′-GGATGTTACCAAGTATTATA**C**TTACCTGTAT AGTGTACTTC-3′,
Y570A: 5′ CTGTCATGGCTTCAG**GC**CTTCAGCATTCC AC -3′

ABCG2 variants were subsequently sequenced (GATC Biotech AG, Cologne, Germany) to verify successful mutation.

### Cell Culture

HEK293-Tet-On cells stably expressing the reverse tetracycline-controlled transactivator (rtTA2^S^-M2) were purchased from Clontech (Cat-No. 630931; Heidelberg, Germany) and cultivated in Dulbecco’s Modified Eagle Medium-High Glucose with 10% fetal bovine serum and 1% penicillin/streptomycin at 37°C with 5% CO_2_.

### Transient Transfection of HEK293 Tet-On Cells

HEK293-Tet-On cells were transiently transfected with the pTRE-Tight-BI-AcGFP1-ABCG2 vectors containing ABCG2 wild-type or the variant sequences, respectively. Transfection was carried out as previously described (Deppe et al., 2014; Ripperger and Benndorf, 2016). For this purpose, HEK293-Tet-On cells were detached by trypsinization and seeded overnight in 6-well plates at a density of 600,000 cells per well. The next day, cells were transiently transfected with 2 μg of the respective DNA/well using the TurboFect reagent (Thermo Fisher Scientific) according to the manufacturer’s recommendations. 24 h after the transfection procedure, HEK293-Tet-On cells were treated for additional 24 h with the tetracycline derivative doxycycline (1 μg/mL) to induce concomitant expression of AcGFP1 as well as ABCG2 wild-type/variants. For PhA efflux assays or flow cytometric analyses of ABCG2 cell surface expression, a part of the transfected HEK293-Tet-On cells were not treated with doxycycline and served as negative control.

### ABCG2 Inhibition Assay (PhA Efflux Assay)

The ABCG2 inhibition assay using PhA as fluorescent ABCG2 substrate was performed as described previously (Weiss et al., 2009; Weiss et al., 2010; Deppe et al., 2014; Ripperger and Benndorf, 2016). In brief, doxycycline-treated and untreated non-induced HEK293-Tet-On cells transiently transfected with the pTRE-Tight-Bl-AcGFP1-ABCG2 wild-type vector or the ABCG2 variant vectors, respectively, were trypsinized, detached, and incubated in incubation medium (RPMI with 2% FCS) containing 1 μM PhA and incubated at 37°C for 30 min on a rotary shaker. Cells were then washed once with ice-cold incubation medium and resuspended in incubation medium containing no (basal efflux) or increasing concentrations (0.1–1000 μmol/L) of the various test substances. After incubation for 60 min at 37°C on a rotary shaker, cells were washed with ice-cold PBS with 2% FCS, resuspended in ice-cold PBS with 2% FCS and kept on ice until flow cytometry. Flow cytometry was performed using an Attune^®^ Acoustic Focusing flow cytometer (Thermo Fisher Scientific). Flow cytometric analysis of median PhA intensity was evaluated in doxycycline-treated AcGFP1-positive HEK293-Tet-On cells (concomitantly expressing ABCG2 wild-type or ABCG2 variants, respectively) as well as in AcGFP1-negative (and ABCG2-negative) cells serving as control for potential non-specific effects of candesartan cilexetil, irbesartan, losartan, telmisartan, or nisoldipine, respectively. The inhibition ratio (a measure of drug-induced ABCG2 inhibition) was calculated as a ratio of the median PhA-associated fluorescence (MF) in AcGFP1-positive (ABCG2 variant or ABCG2 wild-type-expressing) HEK293-Tet-On cells to the MF in AcGFP1-negative (ABCG2-negative) cells in the absence (basal) or presence of increasing concentrations of the respective test drug divided by the basal wild-type efflux ratio (MF ratio of AcGFP1-positive to AcGFP1-negative cells in untreated ABCG2 wild-type-expressing HEK293-Tet-On cells). Each experiment was performed at least in triplicate. The ABCG2 inhibitor nisoldipine (100 μmol/L) served as a positive control.

### Immunofluorescence Staining and Confocal Laser Scanning Microscopy (CLSM)

Subcellular localization pattern of ABCG2 was studied in permeabilized HEK293-Tet-On cells transiently transfected with pTRE-Tight-BI-AcGFP1-ABCG2 wild-type or the ABCG2 variants, respectively, as described previously (Deppe et al., 2014; Ripperger and Benndorf, 2016). ABCG2 as well as nuclei were visualized by an anti-ABCG2 antibody (clone 5D3, BioLegend, San Diego, CA, United States; 1:500) and phycoerythrin (PE)-labeled secondary anti-mouse antibody or Hoechst 33342, respectively. Cells were analyzed with a Nikon A1 confocal microscope System (Nikon, Melville, NY, United States).

### Western Blot Analysis

Western blot analysis of cellular ABCG2 protein levels was performed as previously described (Benndorf et al., 2003, 2008, 2009; Biermann et al., 2012). Proteins were visualized using specific antibodies against ABCG2 (BXP-21, Abcam, Cambridge, United Kingdom) at a dilution of 1:1,000 and AcGFP1 (Clontech; 1:10,000) for normalization.

### Real Time RT-PCR

TaqMan reactions were carried out as described previously (Ripperger and Benndorf, 2016) in 96-well plates according to the manufacturer’s instructions using a pre-made probe for ABCG2 (Hs01053790_m1) and a custom-made (assay ID number AJT96DY, Thermo Fisher Scientific) TaqMan probe for AcGFP1 that served as control. mRNA expression was quantified using the ABI 7500 Real-Time PCR System (Thermo Fisher Scientific). We performed relative quantification of gene expression using the delta-delta CT method (Biermann et al., 2012).

### Flow Cytometric Analyses

Cell membrane-located ABCG2 was analyzed in intact HEK293-Tet-On cells as previously described (Ripperger and Benndorf, 2016). In brief, non-permeabilized ABCG2-overexpressing or control HEK293-Tet-On cells were incubated with an APC-conjugated anti-ABCG2 monoclonal antibody for 20 min for detection of cell surface-located ABCG2 (clone 5D3, BioLegend; 1:50). Afterwards, cells were washed with 1× cell staining buffer (BioLegend) and ABCG2 expression was analyzed using an Attune^®^Acoustic Focusing flow cytometer (Thermo Fisher Scientific). Flow cytometric data were analyzed and plotted using the FlowJo software package (Tree Star Inc., Ashland, TN, United States).

### *In Silico* Homology Modeling and Docking Studies

#### Homology Modeling

Homology models of ABCG2-TDMs were generated using the crystal structure of mouse ABCB1 (PDB ID: 3G5U (Aller et al., 2009) as template as previously described by Rosenberg et al. (Rosenberg et al., 2010). Primary sequence of human ABCG2 was taken from the Universal Protein Resource^1^ (accession code: Q9UNQ0); and the crystal structure of the template was downloaded from the Protein Data Bank^2^. The sequence alignment was generated according to Rosenberg et al. (2010) and final model generation was performed using MODELLER9v11^3^ (Eswar et al., 2006).

#### Docking to Generated Homology Model

Molecular Operating Environment [MOE] (2014) (version 2014.09, Chemical Computing Group, Montreal, QC, Canada) was used to generate the molecular structures of selected ARBs. The ligands were subsequently protonated at physiological pH and energy minimized using the MMFF94x force field in MOE. Docking of the ligands was performed using the program GOLD (version 5.2), while assigning Arg482 as the centroid of the binding site and using Goldscore as a fitness function.

#### Docking to Crystal Structure of ABCG2

Molecular Operating Environment [MOE] (2014) (version 2014.09, Chemical Computing Group, Montreal, QC, Canada) was used to generate the molecular structures of selected ARBs. The ligands were subsequently prepared for docking using the LigPrep tool (LigPrep, 2017) as implemented in Schrödinger’s software, where all possible tautomeric forms as well as stereoisomers were generated and energy minimized using the OPLS force field. Conformers of the prepared ligands were calculated with ConfGen (Watts et al., 2010; ConfGen, 2017) using the default settings (Fast) and allowing minimization of the output conformations.

The structure of ABCG2 in complex with 5D3-Fab (PDB ID: 5NJ3 (Taylor et al., 2017) was retrieved from the Protein Data Bank, and the Fab molecule was deleted. The protein structure encompassing the TMDs and NBDs was subsequently prepared with Schrödinger’s Protein Preparation Wizard (Glide, 2017); where hydrogen atoms were added and the hydrogen bond network optimized. The protonation states at pH 7.0 were predicted using the PROPKA tool in Schrödinger. The structures were finally subjected to a restrained energy minimization step using the OPLS2005 force field (RMSD of the atom displacement for terminating the minimization was 0.3 Å).

The receptor grid preparation for the docking procedure was carried out by assigning Arg482 of chain A as the centroid of the grid box. The generated 3D conformers were docked into the prepared protein structure using Glide (2017) (Schrödinger, Inc., New York City, NY, United States) in the Standard Precision mode. A total of 20 poses per ligand conformer were included in the post-docking minimization step and a maximum of two docking poses were output for each ligand conformer.

### Statistical Analyses

Data were analyzed using GraphPad Prism Version 5.02 (GraphPad Software, San Diego, CA, United States). Statistical analyses were performed using one-way analysis of variance followed by the Fisher‘s protected least significant difference test or the student’s *t*-test as appropriate. Data are given as mean ± standard deviation (SD). Probability values were considered significant at a *P* < 0.05.

## Results

### *In Silico* Homology Modeling and Molecular Interaction Studies

Since the crystal structure of ABCG2 was not available when we started our *in silico* studies on the interaction of ARBs with ABCG2, we used homology modeling with mouse Abcb1 as a template to predict critical residues for this interaction. The sequence alignment of TMDs of ABCG2 to mouse ABCB1 showed a very poor sequence identity of 15.8%, a sequence similarity of 25.7% and was characterized by the presence of various long gaps. We were well aware that the prerequisite for the generation of a reliable homology model were not present, however, no better templates were available at that time, which prompted us to rely on homology models for subsequent molecular docking as well as mutation studies.

**FIGURE 1:**
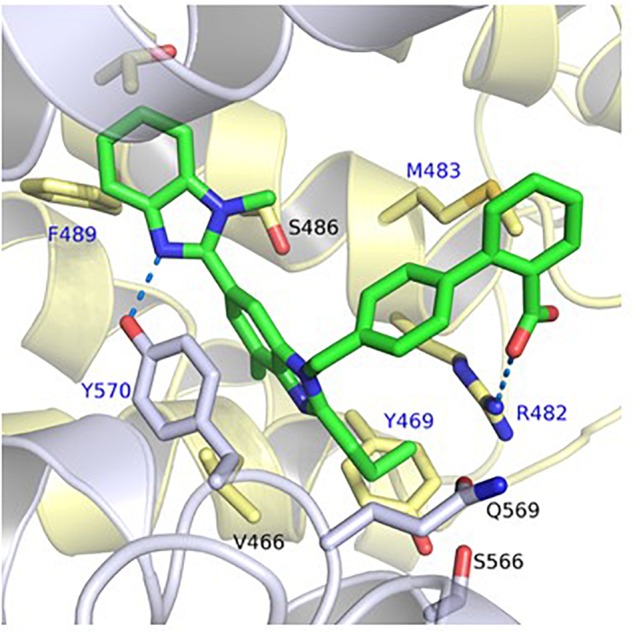
Predicted binding mode of telmisartan in the generated homology model of ABCG2. The first ABCG2 monomer is colored in yellow, the second monomer in white, and the ligands in green. Hydrogen bond and salt bridge interactions are shown as blue-dashed lines. The amino acids which were mutated in this study are marked by a blue label.

The predicted binding mode of telmisartan in the generated homology model of ABCG2 is shown in **Figure 1**. This suggested that the carboxylate group of telmisartan undergoes salt bridge interactions with Arg482 of the first ABCG2 monomer (G1) while its phenyl group shows hydrophobic interactions with Met483 of G1. The benzimidazole core is embedded in a hydrophobic pocket surrounded by Tyr469 and Val466 of G1 as well as Tyr570 of the second ABCG2 monomer (G2). Meanwhile, the other benzimidazole moiety shows hydrogen bond interactions with Tyr570 of G2 and hydrophobic interactions with Phe489 of G1. Based on this suggested binding mode, Arg482, Phe489, Tyr469, Met483, and Tyr570 were predicted to affect binding of ARBs to ABCG2.

### Impact of ABCG2 Mutations on ABCG2 mRNA Expression and Cellular Protein Content as Well as Subcellular Localization of the ABC Transporter

**FIGURE 2:**
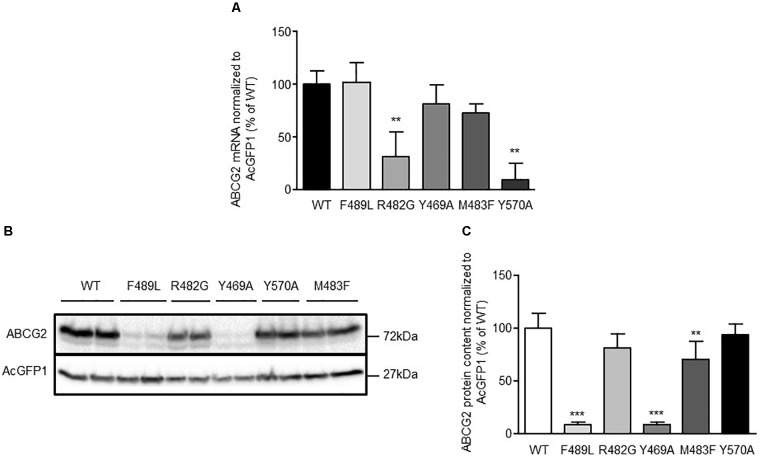
**(A)** Analysis of ABCG2 mRNA expression in transiently transfected HEK293-Tet-On cells. Data are shown as mean ± SD (*n* = 3–6), the mean of ABCG2 wild-type (WT) was arbitrarily set to 100%. ^∗∗^*P* < 0.01 vs. ABCG2 WT. **(B)** Representative Western Blot and **(C)** densitometric analysis of ABCG2 protein content in HEK293-Tet-On cells transiently transfected with ABCG2 WT or ABCG2 variants, respectively. Data are expressed as mean ± SD (*n* = 5), the mean of ABCG2 WT was arbitrarily set to 100%. ^∗∗^*P* < 0.01 vs. ABCG2 WT. ^∗∗∗^*P* < 0.001 vs. ABCG2 WT.

**FIGURE 3:**
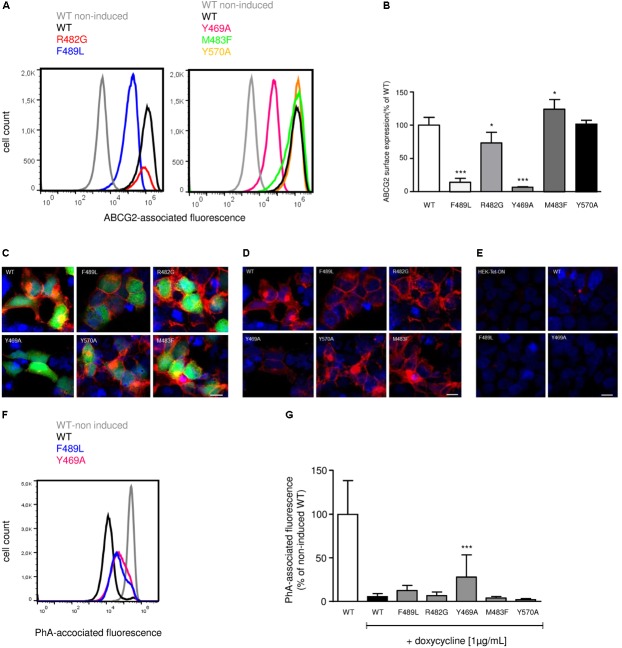
**(A)** Representative flow cytometric plots of cell surface-related ABCG2 fluorescence intensity of non-permeabilized, either untreated (non-induced) or doxycycline-treated (1 μg/mL; 24 h) HEK293-Tet-On cells transiently transfected with ABCG2 WT or ABCG2 variants, respectively. **(B)** Flow cytometric analysis of plasma membrane-related ABCG2 fluorescence intensity in non-permeabilized HEK293-Tet-On cells transiently transfected with ABCG2 WT or ABCG2 variant vectors, respectively. Data are shown as mean ± SD (*n* = 3–18), the mean of ABCG2 WT was arbitrarily set to 100%. ^∗^*P* < 0.05 vs. WT. ^∗∗∗^*P* < 0.001 vs. WT. **(C,D)** Evaluation of the subcellular localization pattern of ABCG2 WT or ABCG2 variants in permeabilized HEK293-Tet-On cells using confocal laser scanning microscopy. ABCG2 (red), nuclei (Hoechst 33342, blue) and the green fluorescent protein (AcGFP1, green) were visualized in doxycycline-treated (induced) HEK293-Tet-On cells transiently transfected with ABCG2 WT or ABCG2 variants, respectively. **(C)** shows merged ABCG2-, AcGFP1-, and Hoechst 33342-related fluorescence, whereas in **(D)** the AcGFP1 signal has been excluded to highlight ABCG2-related fluorescence. **(E)** ABCG2 is not detectable in native (non-transfected) HEK293-Tet-On cells or in doxycycline-naïve (non-induced) HEK293-Tet-On cells transfected with ABCG2 WT or ABCG2 variants Y469A or F489L, respectively. White scale bar indicates 50 μm. **(F)** Representative flow cytometric plots of PhA-associated fluorescence intensity in non-induced control cells (gray) or in doxycycline-treated HEK293-Tet-On cells transiently transfected with the ABCG2 WT (black) or ABCG2 variants Y469A (pink) or F489L (blue), respectively. **(G)** PhA efflux assay to evaluate ABCG2 activity in transiently transfected HEK293-Tet-On cells. PhA efflux was analyzed in either untreated or doxycycline-treated (1 μg/mL; 24 h) HEK293-Tet-On cells transiently transfected with ABCG2 WT or ABCG2 variant vectors, respectively. Data are shown as mean ± SD (*n* = 8–38), the mean of non-induced ABCG2 WT-transfected HEK293-Tet-On cells was arbitrarily set to 100%. ^∗∗∗^*P* < 0.001 vs. induced WT.

To elucidate the impact of ABCG2 mutations on ABCG2 mRNA and protein expression, mRNA and proteins were extracted from doxycycline-treated (1 μg/mL; 24 h) HEK293-Tet-On cells transiently transfected with pTRE-Tight-Bl-AcGFP1-ABCG2 wild-type and ABCG2 variants, respectively. To ensure comparability, all genotypes were analyzed simultaneously within the same set of experiments (transfection and induction procedure, reverse transcription and real-time PCR analyses, Western Blot-, flow cytometric-, and microscopic analyses). As shown in **Figure 2A**, mRNA expression of cells transfected with the R482G (31.1 ± 23.7%; *n* = 6; *P* < 0.01 vs. ABCG2 wild-type) and Y570A (9.9 ± 15.0%; *n* = 3; *P* < 0.01 vs. ABCG2 wild-type) variants, respectively, were significantly lower than mRNA expression of ABCG2 wild-type-transfected cells (100 ± 12.4%; *n* = 6). As indicated in **Figures 2B,C**, ABCG2 protein levels of cells transfected with the Y469A (8.7 ± 2.9%; *n* = 5; *P* < 0.001 vs. ABCG2 wild-type) and the F489L (9.1 ± 2.1%; *n* = 5; *P* < 0.001 vs. ABCG2 wild-type) variants, respectively, were lower than those transfected with ABCG2 wild-type (100 ± 14.6%; *n* = 5). In flow cytometric analyses, a similar reduction in ABCG2 cell surface expression (**Figures 3A,B**) was observed in cells transfected with the Y469A (6.6 ± 0.2%; *n* = 3; *P* < 0.01 vs. ABCG2 wild-type) and the F489L (14.1 ± 6.0%; *n* = 15; *P* < 0.01 vs. ABCG2 wild-type) variants, respectively, as compared with ABCG2 wild-type-transfected cells (100 ± 11.7%; *n* = 15). Nevertheless, ABCG2 wild-type and all investigated variants were primarily located at the plasma membrane as evidenced by confocal laser scanning microscopy (**Figures 3C,D**). In contrast, ABCG2-related fluorescence was not detectable in native HEK293-Tet-On cells or in non-induced HEK293-Tet-On cells transfected with ABCG2 WT or ABCG2 variants Y469A or F489L, respectively (**Figure 3E**).

### Impact of ABCG2 Mutations on ABCG2-Mediated Basal Pheophorbid A (PhA) Transport and ARB-Induced Inhibition of ABCG2-Mediated PhA Efflux

Non-induced (AcGFP1-and ABCG2-negative) transfected HEK293-Tet-On cells served as control for potential non-specific effects of ARBs, respectively, and as indicator of baseline cellular PhA accumulation in the absence of relevant ABCG2 expression. For the analysis of basal PhA efflux, the averaged median PhA-associated fluorescence of non-induced HEK293-Tet-On cells transiently transfected with the pTRE-Tight-Bl-AcGFP1-ABCG2 wild-type vector was arbitrarily set to 100 ± 38.6% (*n* = 11). To analyze the impact of doxycycline-induced ABCG2 expression on cellular PhA accumulation, median PhA-associated fluorescence was selectively analyzed in AcGFP1-positive (and hence ABCG2-expressing) HEK293-Tet-On cells (**Figures 3F,G**). Induction of ABCG2 wild-type expression reduced PhA-associated fluorescence to (5.4 ± 3.5%; *n* = 34) of non-induced ABCG2 wild-type-transfected cells, demonstrating expression of functional ABCG2 in AcGFP1-positive cells (**Figures 3F,G**). PhA transport was similar in doxycycline-induced AcGFP1-positive HEK293-Tet-On cells transfected with the ABCG2 variants R482G (6.6 ± 4.1%; *n* = 22) and M483F (3.9 ± 1.8%; *n* = 26) and tended to be increased in Y570A-transfected (2.0 ± 1.2%; *n* = 8) cells. In contrast, basal PhA efflux was significantly impaired in HEK293-Tet-On cells transfected with the ABCG2 variant Y469A (27.9 ± 25.6%; *n* = 38; *P* < 0.001 vs. doxycycline-induced ABCG2 wild-type) and tended to be lower in F489L-transfected cells (12.4 ± 5.9%; *n* = 22).

**FIGURE 4:**
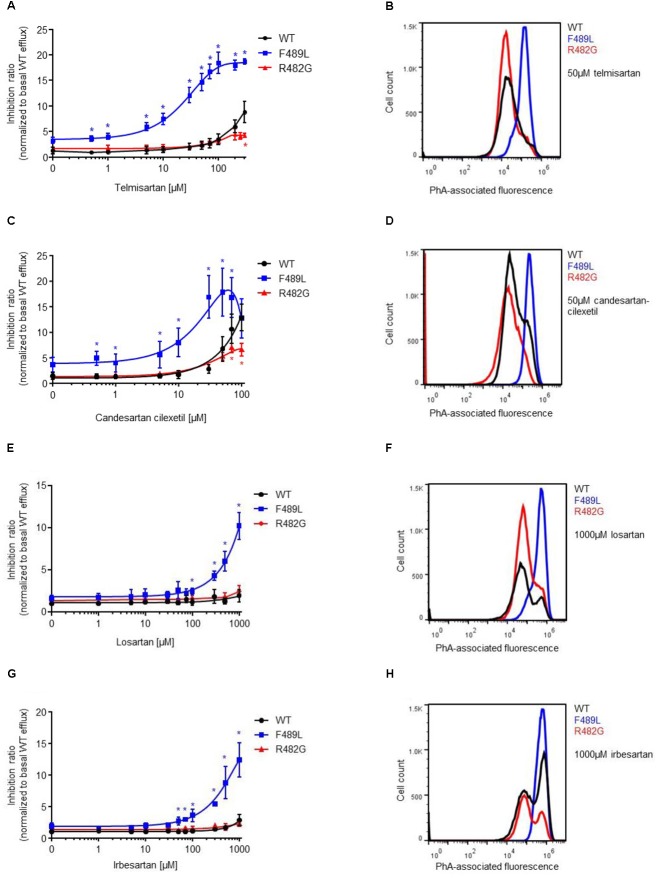
PhA efflux assay: concentration-dependent inhibition of ABCG2-mediated PhA efflux by telmisartan **(A,B)**, candesartan cilexetil **(C,D)**, losartan **(E,F)**, and irbesartan **(G,H)** in HEK293-Tet-On cells transiently transfected with ABCG2 WT or ABCG2 variants (F489L and R482G), respectively. Data are shown as mean ± SD (*n* = 4–16). ^∗^*P* < 0.05 vs. WT. Representative flow cytometric plots of genotype-specific telmisartan- **(B)**, candesartan cilexetil- **(D)**, losartan- **(F)**, and irbesartan **(H)**-related inhibition of ABCG2-mediated PhA efflux.

**FIGURE 5:**
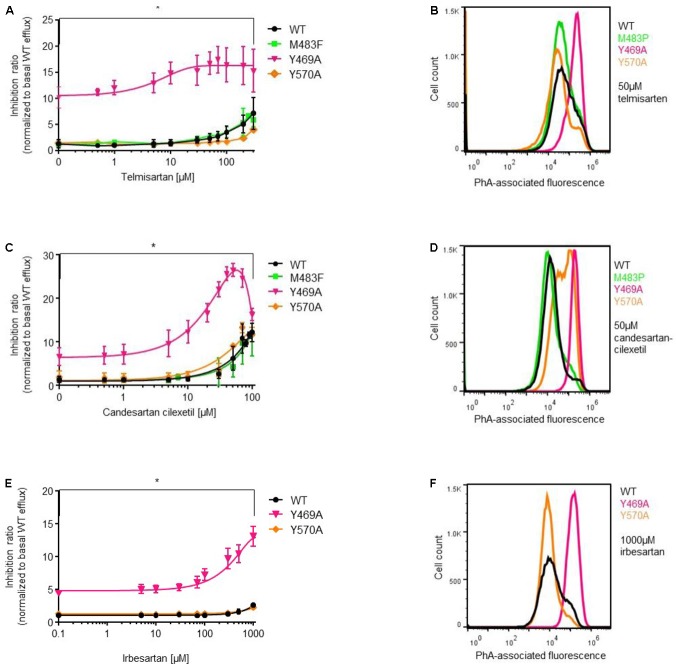
PhA efflux assay: concentration-dependent inhibition of ABCG2-mediated PhA efflux by telmisartan **(A,B)**, candesartan cilexetil **(C,D)**, and irbesartan **(E,F)** in HEK293-Tet-On cells transiently transfected with ABCG2 WT or ABCG2 variants (Y469A, M483F, and Y570A), respectively. Data are shown as mean ± SD (*n* = 4–14). ^∗^*P* < 0.05 vs. WT. Representative flow cytometric plots of genotype-specific telmisartan- **(B)**, candesartanclexetil- **(D)**, and irbesartan **(F)**-related inhibition of ABCG2-mediated PhA efflux.

Next, we analyzed the impact of ABCG2 mutations on ARB-induced inhibition of PhA efflux in transiently transfected HEK293-Tet-On cells. As expected, candesartan cilexetil and telmisartan concentration-dependently inhibited PhA efflux in ABCG2 wild-type-transfected cells (**Figures 4**, **5**). However, candesartan cilexetil- and telmisartan-induced inhibition of PhA efflux did not reach a plateau within the chosen (non-toxic) concentration range in HEK293-Tet-On cells transiently transfected with ABCG2 wild-type so that comparative (genotype-related) IC_50_ calculations were not possible. Inhibitory characteristics of candesartan cilexetil and telmisartan were not significantly altered in cells transfected with the ABCG2 variants M483F and Y570A (**Figures 5A,C**) but were significantly attenuated in cells transfected with the ABCG2 variant R482G (**Figures 4A,C**), thereby indicating that the arginine residue at position 482 of the ABCG2 molecule may be of importance for the interaction of both ARBs with the ABC transporter. In contrast, the F489L polymorphism enhanced both candesartan cilexetil and telmisartan-induced inhibition of ABCG2-related PhA transport in our experimental system (**Figure 4**). As described previously (Weiss et al., 2010), candesartan cilexetil concentrations of 100 μmol/L and higher induced cytotoxic effects in HEK293 cells (data not shown) that may explain the drop of inhibition ratio observed at higher concentrations of this ARB. Moreover, high concentrations (100 μmol/L and higher) of irbesartan and losartan both significantly inhibited the PhA efflux capacity of cells transfected with the F489L variant, whereas they did not relevantly affect PhA transport of ABCG2 wild-type-transfected cells in this setting (**Figure 4**), thus indicating that the F489L polymorphism may in general facilitate the inhibitory interaction of these ARBs with ABCG2. In addition, the Y469A substitution induced a very similar ABCG2 phenotype and enhanced the inhibitory interaction of candesartan cilexetil, irbesartan and telmisartan with the ABC transporter (**Figure 5**).

### Molecular Interaction Studies on Newly Released ABCG2 Crystal Structure

The newly released structure (Taylor et al., 2017) clearly reveals that ABCG2 possesses a distinct fold from that found in the mouse ABCB1, which was used as a template for the generation of the homology model. Interestingly, the transmembrane helices and intracellular loops of ABCG2 are much shorter. The generated homology model was, thus, not accurate and rather unreliable. We, therefore, used the crystal structure to perform further molecular docking studies of ARBs and to try and rationalize the herein obtained results of mutation studies.

**FIGURE 6:**
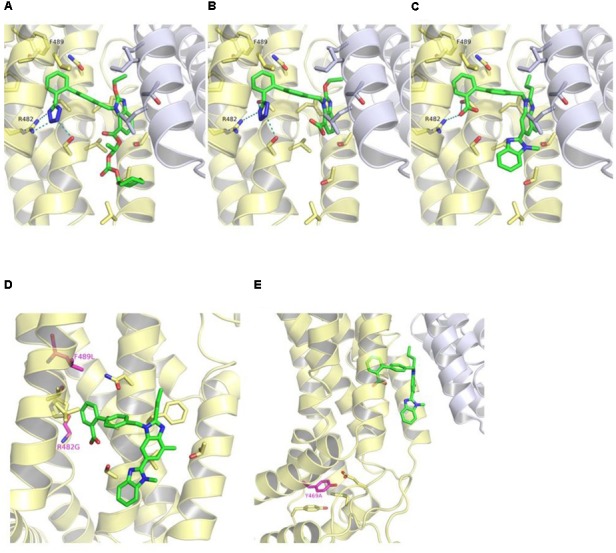
Binding mode of candesartan cilexetil **(A)**, candesartan **(B)**, and telmisartan **(C)** in the crystal structure of ABCG2 (PDB ID: 5NJ3) as predicted by docking studies. The first ABCG2 monomer is colored in yellow, the second monomer in white, and the ligands in green. Hydrogen bond and salt bridge interactions are shown as teal-dashed lines. R482G and F489L substitutions (purple) **(D)** and location of Tyr469 (purple) in ABCG2 crystal structure **(E)**. The predicted binding mode of telmisartan is shown as green sticks in **(D,E)**.

Docking studies of ARBs, as exemplified by telmisartan, candesartan cilexetil, and hydrolytic product candesartan (**Figure 6**), revealed that the hydrophobic alkyl/alkyloxy-benzimidazole core is embedded in the hydrophobic cavity that is formed at the TMD interface between TM2 and TM5a of opposing ABCG2 monomers, and which was has been ascribed the role as the substrate-binding pocket (Taylor et al., 2017). The cilexetil moiety of candesartan cilexetil is accommodated in the lower pocket at this interface between TM2 and TM5a. The terminal phenyl moiety is accommodated in a hydrophobic subpocket (site 2) lined by Val401 of TM1, Asn436 of TM2, as well as Pro485 and Phe489 of TM3. Meanwhile, the electronegative carboxylate and tetrazole groups of telmisartan, candesartan cilexetil, and candesartan, respectively, occupy an electropositive region formed by Gln398 of TM1, Ser440, and Ser443 of TM2 as well as Arg482 of TM3, where they are able to undergo salt bridge and hydrogen bond interactions.

We also examined the positions of the herein reported variations in the released structure of ABCG2 (**Figures 6D,E**). Phe489 is located in site 2, which is part of the predicted ARBs binding pocket. It is possible, that the F489L polymorphism may induce conformational changes within site 2 and, hence, modulate the binding affinities of ARBs. Meanwhile, Arg482 seems to play a key role in the binding of ARBs to ABCG2 through the formation of a salt bridge interaction with the respective carboxylate or tetrazole groups. Docking of ARBs to the R482G variant gave no consistent binding modes (data not shown).

On the other hand, Tyr469 is located at the bottom of TM3 far off the substrate-binding pocket and the predicted binding site of ARBs. Tyr469 undergoes π-π stacking interactions with Tyr464 of L2 and Phe455 of TM2 as well as hydrogen bond interactions with Glu451 of TM2 (**Figure 6**). The Y469A substitution might, hence, have an impact on the proper folding of the protein.

## Discussion

AT1 receptor antagonists (ARB) are a widely prescribed group of antihypertensive drugs that also play an increasing role in the pharmacotherapeutic management of heart failure and diabetic nephropathy. Moreover, they are important components of the recently approved drug class of angiotensin receptor neprilysin inhibitors (ARNi) that combine pharmacological neprilysin inhibition with an ARB moiety for the effective treatment of chronic heart failure with reduced ejection fraction (Yancy et al., 2016). As ARBs are often prescribed as a part of multi-drug regimens in these patients, they may also cause drug-drug interactions that compromise drug safety and efficacy. In this regard, we have demonstrated previously that – within the class of ARBs – in particular telmisartan and the prodrug candesartan cilexetil and to a minor extent also irbesartan and losartan were able to inhibit the activity of ABC transporters, such as ABCB1, ABCC2, and ABCG2, *in vitro* (Weiss et al., 2010) and may thus affect the disposition of further drugs *in vivo* (Stangier et al., 2000; Bajcetic et al., 2007; Son et al., 2014). Moreover, we have previously shown that the interaction of telmisartan with ABCG2 is influenced by naturally occurring variations within the ABCG2 gene (Deppe et al., 2014). In this context, we observed that the non-synonymous F489L polymorphism increased, whereas the R482G substitution blunted the inhibitory interaction of telmisartan with the ABC transporter. These data lead us to assume that this polymorphism could be of general relevance for the interaction of ARBs with the ABC transporter. Thus, a major aim of the present study was to elucidate whether the F489L polymorphism and the R482G variation may also affect the interaction of further ARBs previously shown to reduce the efflux capacity of ABCG2 *in vitro* (Weiss et al., 2010) with the efflux transporter.

As reported before, we observed in the present study a F489L-associated reduction in cellular ABCG2 protein (but not mRNA) abundance in HEK293 cells that may be related to an enhanced proteasomal degradation of the aberrant protein (Deppe et al., 2014). These observations are in accordance with experimental findings from further groups (Itoda et al., 2003; Kobayashi et al., 2005; Sjostedt et al., 2017) and may have clinical implications in that reduced ABCG2 protein levels in individuals carrying the F489L polymorphism could affect the pharmacokinetic profile of ABCG2 substrates, i.e., statins such as rosuvastatin, and hence, increase the likelihood of adverse drug reactions. Nonetheless, this reduction in ABCG2 expression did not result in a significant impairment in ABCG2-related PhA efflux in our system, thereby suggesting that the observed reduction in ABCG2 protein content may be compensated by a more efficient PhA transport by the aberrant protein or that protein content may not be linearly linked to ABCG2-dependent substrate transport in ABCG2 overexpressing cells *in vitro*.

Furthermore, we observed that the F489L polymorphism enhanced the inhibitory effect of candesartan cilexetil and telmisartan on ABCG2-related PhA efflux apparently in terms of inhibitory potency (although comparative IC_50_ calculations were not possible because ARB-induced inhibition of ABCG2 PhA efflux function did not reach a plateau within the chosen non-toxic concentration range). In contrast, the R482G variation blunted the inhibitory effect of both ARBs in this context. Moreover, the F489L polymorphism uncovered the inhibitory effect of both irbesartan and losartan on ABCG2-mediated PhA efflux, whereas neither irbesartan nor losartan relevantly affected the activity of wild-type or R482G-mutated ABCG2 in this setting. Thus, our data suggest that the F489L polymorphism, a polymorphism that has been described to occur with rather low prevalence in Asian individuals (Itoda et al., 2003), in general could render ABCG2 susceptible toward the inhibitory effects of ARBs and in the clinical setting may identify individuals at high risk of developing ARB-induced and ABCG2-related drug-drug interactions *in vivo*. The reason for this phenomenon remains unclear, but it is tempting to speculate that the F489L polymorphism may induce conformational changes within the ARB binding pocket, specifically in site 2 which is formed between TM1, TM2, and TM3. In agreement with this concept, our molecular docking studies on newly released ABCG2 crystal structure (Taylor et al., 2017) as well as recently published studies using an ABCG2 homology model (based on structural information of the human ABCG5-ABCG8 heterodimer (Lee et al., 2016) indicated that this hydrophobic site (site 2) may be involved in the formation of ARBs binding pocket and participate in the transport of drugs and its metabolites (Laszlo et al., 2016). Furthermore, our studies suggested that Arg482 is crucial for the binding of ARBs through the formation of a salt bridge interaction with the negatively charged carboxylate or tetrazole groups. Mutation of Arg482 might, hence, attenuate the binding of ARBs to ABCG2. In line with this notion, the somatic mutation R482G, a mutation that frequently occurs in drug-resistant cancer cells, has been associated with an altered ABCG2 substrate spectrum, e.g., with respect to cytotoxic drugs (Sarkadi et al., 2006). In example, it has been described that the R482G substitution negatively affects ABCG2-dependent transport of the dihydrofolate reductase inhibitor methotrexate, whereas it acts as a gain-of-function mutation with regard to doxorubicin (Chen et al., 2003; Sarkadi et al., 2006). Thus, our findings not only add to the available evidence on the important role of Arg482 in governing the substrate specificity of ABCG2 but also point to a role of adjacent amino acids (e.g., Phe489) in this matter.

To further explore the structural basis of the ARB-ABCG2 interaction, we developed an ABCG2 homology model based on available structural information derived from a mouse ABCB1 analog (Deppe et al., 2014) (that was the closest homologue at the time we started with these *in silico* studies) and subsequently performed docking analyses to identify amino acids with potential relevance for the ARB-ABCG2 interaction. Afterwards, we experimentally validated the impact of several amino acid substitutions (Y469A; M483F; Y570A) derived from these *in silico* predictions on the interaction of telmisartan as well as irbesartan and candesartan cilexetil with the ABC transporter. Using this approach, we could first show that the Y469A substitution severely reduced the ABCG2 protein content in HEK293 cells and significantly impaired ABCG2-related PhA efflux capacity. Interestingly, the Y469A substitution apparently also increased the inhibitory potency of all tested ARBs on ABCG2-mediated PhA efflux and thereby mimicked the ABCG2 phenotype induced by the F489L polymorphism. Tyr469 is located at the bottom of TM3 showing π-π stacking interactions with Tyr464 of L2 and Phe455 of TM2 as well as hydrogen bond interactions with Glu451 of TM2. The Y469A substitution might, hence, impact the folding of the protein.

In contrast, neither the M483F nor the Y570A substitution relevantly affected cellular ABCG2 protein content or PhA efflux function of the transporter in our study. Moreover, neither of these two substitutions had a significant impact on the interaction of candesartan cilexetil, irbesartan, or telmisartan with the ABC transporter. These data prove that our mouse ABCB1-related ABCG2 homology model (and predictions of drug-protein interactions deduced therefrom) were strongly hampered by low sequence homology and considerable differences with respect to membrane topology, i.e., transmembrane helix arrangements, of mouse ABCB1 and ABCG2, an issue that has been observed and addressed previously (Chen et al., 2003). Indeed, recapitulation of the newly released ABCG2 structure (Taylor et al., 2017) indicated that Met483 and Tyr570 (in contrast to Tyr469) are surface exposed and play no role in protein folding or substrate binding.

In summary, our work suggests that the amino acid sequence near the third transmembrane helix of ABCG2 are of importance for the interaction of ARBs with the ABC transporter. Moreover, our data indicate that individuals carrying the F489L polymorphism may be at increased risk of developing ABCG2-dependent drug-drug interactions in multi-drug regimens involving ARBs and ABCG2 substrates and that, thus, further investigations could be helpful to evaluate the significance of these *in vitro* findings in the clinical context.

## Author Contributions

RB, AR, DR, and WS designed the research study and wrote the manuscript. AR, AK, and DR performed the experiments. AR, AK, DR, WS, and RB analyzed the results.

## Conflict of Interest Statement

The authors declare that the research was conducted in the absence of any commercial or financial relationships that could be construed as a potential conflict of interest.

## Footnotes

1 http://www.uniprot.org

2 http://www.rcsb.org/

3 https://salilab.org/modeller/9.11/release.html

## Supplementary Material

FIGURE S1 Schematic illustration of the pTRE-Tight-BI-AcGFP1-ABCG2 vector structure.
