# Supplementary figures and images for: Pharmacogenetic Aspects of the Interaction of AT1 Receptor Antagonists With ATP-Binding Cassette Transporter ABCG2
Source: Front Pharmacol. 2018 May 14;9:463. doi: 10.3389/fphar.2018.00463 (PMC5960723; doi:10.3389/fphar.2018.00463)

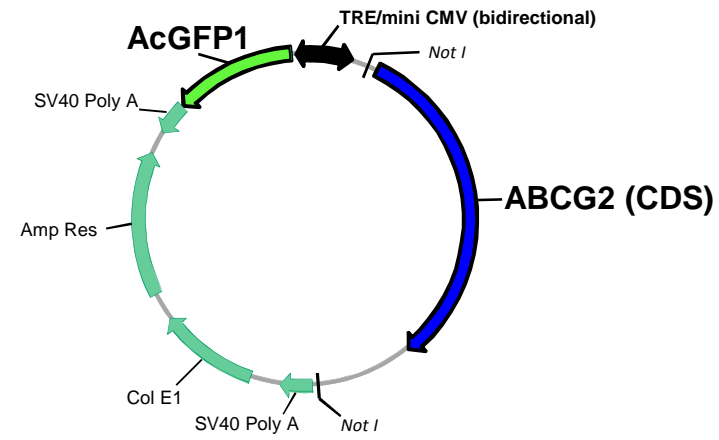

Supplement: FIGURE S1 — Schematic illustration of the pTRE-Tight-BI-AcGFP1-ABCG2 vector structure. [file Image_1.PDF]
